# Ischemia considerations for the development of an organ and tissue donor derived bone marrow bank

**DOI:** 10.1186/s12967-020-02470-1

**Published:** 2020-08-05

**Authors:** Erik J. Woods, Aubrey M. Sherry, John R. Woods, James W. Hardin, Michael LaFontaine, Gerald Brandacher, Brian H. Johnstone

**Affiliations:** 1 Ossium Health, Inc., 5742 W. 74th St, Indianapolis, IN 46278 USA; 2 Department of Biomedical Sciences, College of Osteopathic Medicine, Marian University, 3200 Cold Spring Rd, Indianapolis, IN 46222 USA; 3 Department of Medical and Molecular Genetics, Indiana University School of Medicine, 975 W. Walnut St., Medical Research Library Building, IB 130, Indianapolis, IN 46202 USA; 4 Richard M. Fairbanks School of Public Health, Indiana University, 1050 Wishard Blvd, Indianapolis, IN 46202 USA; 5 Arnold School of Public Health, University of South Carolina, 921 Assembly St, Columbia, SC 29208 USA; 6 Department of Plastic and Reconstructive Surgery, Johns Hopkins University School of Medicine, 601 N. Caroline St, Baltimore, MD 21231 USA

**Keywords:** Deceased-donor bone marrow, Bone marrow banking, Bone marrow ischemia time, Hematopoietic stem cell transplant

## Abstract

**Background:**

Deceased organ donors represent an untapped source of therapeutic bone marrow (BM) that can be recovered in 3–5 times the volume of that obtained from living donors, tested for quality, cryopreserved, and banked indefinitely for future on-demand use. A challenge for a future BM banking system will be to manage the prolonged ischemia times that are inevitable when bones procured at geographically-dispersed locations are shipped to distant facilities for processing. Our objectives were to: (a) quantify, under realistic field conditions, the relationship between ischemia time and the quality of hematopoietic stem and progenitor cells (HSPCs) derived from deceased-donor BM; (b) identify ischemia-time boundaries beyond which HSPC quality is adversely affected; (c) investigate whole-body cooling as a strategy for preserving cell quality; and (d) investigate processing experience as a variable affecting quality.

**Methods:**

Seventy-five bones from 62 donors were analyzed for CD34+ viability following their exposure to various periods of warm-ischemia time (WIT), cold-ischemia time (CIT), and body-cooling time (BCT). Regression models were developed to quantify the independent associations of WIT, CIT, and BCT, with the viability and function of recovered HSPCs.

**Results:**

Results demonstrate that under “real-world” scenarios: (a) combinations of warm- and cold-ischemia times favorable to the recovery of high-quality HSPCs are achievable (e.g., CD34+ cell viabilities in the range of 80–90% were commonly observed); (b) body cooling prior to bone recovery is *detrimental to* cell viability (e.g., CD34+ viability < 73% *with*, vs. > 89% *without* body cooling); (c) vertebral bodies (VBs) are a superior source of HSPCs compared to ilia (IL) (e.g., %CD34+ viability > 80% when VBs were the source, vs. < 74% when IL were the source); and (d) processing experience is a critical variable affecting quality.

**Conclusions:**

Our models can be used by an emerging BM banking system to formulate ischemia-time tolerance limits and data-driven HSPC quality-acceptance standards.

## Background

Deceased-donor bone marrow (BM) represents a large, untapped source of hematopoietic stem and progenitor cells (HSPCs) that could be cryopreserved and banked for future on-demand use in bone marrow transplant (BMT) procedures. The appeal of BM banking arises in part from the recognition that HSPCs could be immediately available during surges in demand, as, for example, following a mass casualty event such as a nuclear disaster resulting in widespread bone-marrow failure [1, 2]. Interest has been further solidified by recent advances in the induction of immune tolerance via the infusion of donor BM cells to establish durable or transient mixed chimerism and/or peripheral immunomodulation in recipients of solid organ and vascular composite allograft (VCA) transplants [3–5]. A donor BM bank would provide a repository for future tolerance induction and immunomodulation procedures that use delayed protocols. Such procedures have already been proven successful in non-human primates [6, 7].

Cryopreservation and banking of BM from deceased organ donors will require the establishment of BM banks, similar in concept to umbilical cord blood banks. As with cord blood, it is well-established that BM remains biologically functional following cryopreservation and can serve as a genetically diverse, on-demand source of stem cell grafts [8–11]. The national Organ Procurement Organization (OPO) network, which has been active in the United States (US) for over 50 years, provides an existing, well-functioning infrastructure for procuring and transporting bone tissue recovered from deceased donors. However, organizing an integrated organ-donor BM procurement and banking system that capitalizes on this infrastructure, will require coordinated efforts, involving the recovery and safe shipment of biological material to specialized BM cell-processing centers appropriately scaled for clinical production.

A critical issue, which typically has not been viewed as significant in the case of living BM donors, is the management of the prolonged ischemia times that are inevitable during recovery and shipment of bones recovered from deceased donors. Before a clinical production system can be brought to scale, it will be necessary to determine how variations in warm- and cold-ischemia times influence the quality of HSPCs derived from bones recovered at geographically dispersed locations, and shipped long distances to centralized processing facilities. And it will be necessary to establish upper tolerance limits for both warm- and cold-ischemia, which, if exceeded, would likely render the quality and functionality of HSPCs unacceptable for therapeutic use.

Additionally, the impact of whole-body cooling in the context of deceased-donor bone recovery needs to be better understood. Current tissue-banking guidelines in the US allow tissues to be recovered from deceased donors up to 24 h following asystole, provided the body is refrigerated within 12 h of cardiac arrest [12]. However, body cooling is a variable that has not been investigated systematically in relation to the recovery of BM, and it is one that may require different criteria than those established for tissue recovery.

Here we present our results for the first time, which quantify the associations of ischemia time and whole-body cooling, with the quality of HSPCs recovered from the vertebral bodies and ilia of deceased organ donors. Our analyses show that high-quality, functional HSPCs can be obtained from deceased donors even after recovered bones are subjected to cumulative warm- and cold-ischemia times exceeding 40 h, provided that body cooling, which is shown to be detrimental to viability, is avoided. These findings should be useful in establishing industry standards for warm- and cold-ischemia-time tolerance limits and HSPC quality acceptance criteria for BM derived from deceased organ donors.

## Materials and methods

### Study design

This is a pragmatic [13] observational field study designed to model the effects of ischemia and body-cooling times on the viability and function of HSPCs recovered from the BM of deceased organ donors. To assure the study’s external validity (generalizability) we secured the participation of multiple OPOs operating under normal field conditions. Except for special training related to the details of bone recovery and shipment (see below), usual procurement conditions were in effect. Because the OPOs were geographically dispersed, the collected data cover the full spectrum of ischemia times likely to be seen under “real-world” procurement and shipping scenarios. Recovered bones were shipped to one of two processing facilities located in Centennial, CO (Facility A) or Indianapolis, IN (Facility B).

### Donor tissue procurement and transport

Previously developed clinical recovery methods combined with subsequent experience in the ongoing VCA transplant immunomodulation clinical trial at Johns Hopkins University (ClinicalTrials.gov Identifier: NCT01459107) formed the basis for the procurement and transport protocols [4, 14–16]. However, these protocols required optimization and validation to ensure that multiple OPOs could reliably operationalize them in a manner that allowed for the production of consistent yields of functionally viable HSPCs after cross-country transport of recovered bones. To that end, a streamlined OPO recovery procedure, combined with dedicated kits and centralized training on recovery and shipment were employed.

Vertebral sections from T-8 through L-5 (Facility A and B) and/or ilia (Facility A, only) were procured by six OPOs: Gift of Hope (Itasca, IL); Donor Alliance (Denver, CO); Iowa Donor Network (North Liberty, IA); Mid America Transplant (St. Louis, MO); and Nevada Donor Network (Las Vegas, NV). Bones were recovered by OPO personnel using an osteotome and mallet under an IRB approved protocol from research-consented organ and tissue donors. Unprocessed bones were wrapped in lap sponges and towels soaked in saline and placed in triple-sealed bags to ensure moisture retention during shipment. Wrapped specimens were shipped overnight on wet ice.

### Manual debriding

**Fig. 1:**
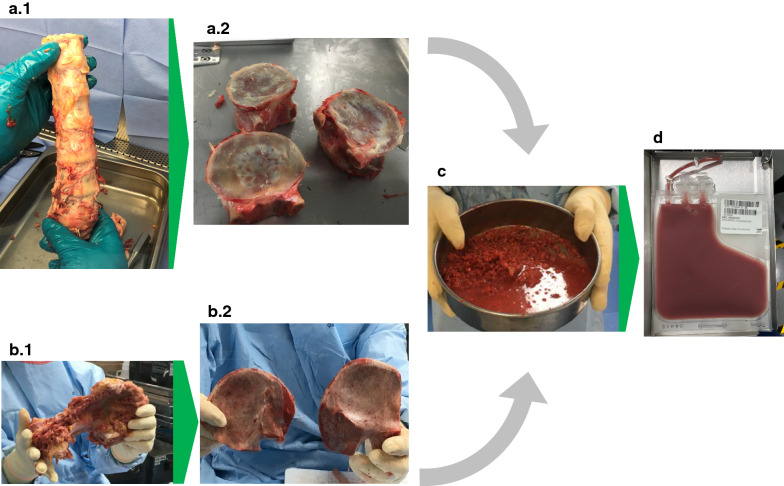
Source material and processing steps for production of deceased-donor derived bone marrow. **a.1** a block of vertebral bodies prior to debriding and disarticulation; **a.2** cleaned and disarticulated vertebral bodies ready for processing; **b.1** a hemipelvis ready for debriding; **b.2** Ilium debrided and ready for processing; **c** cut and ground bone ready for marrow extraction (bones were processed independently); **d** processed bone marrow in final packaging

Upon receipt, in an ISO 5 clean room (Facility A) or a Biological Safety Cabinet (Facility B), soft tissue was manually debrided using scalpels and gouges. Once visible, the pedicles were removed using either a tissue processing band saw or a Stryker System 6 Saw (Stryker, Kalamazoo, MI) leaving only the connected vertebral bodies. Using a boning knife (Facility B) or tissue processing band saw (Facility A), vertebral bodies were separated at the intervertebral disc. Remaining intervertebral disc and soft tissue were removed with a scalpel, leaving clean, separated VBs. Ilium soft tissue was removed with gouges and a scalpel. Care was taken to ensure that the cortical bone was not breached to preserve and protect the hypoxic cancellous BM throughout the entire debriding process (Fig. 1).

Using a saw and/or anvil shears, VBs and IL were cut into 5 cm^3^ pieces small enough for fragmenting with a bone grinder. The pieces were immediately submerged into 500 mL processing medium (Iscove’s Modified Dulbecco’s Medium containing 100 U/mL DNase, 10 U/mL heparin, and 2.5% human serum albumin). IMDM is suitable for rapidly proliferating high-density cell cultures and ideal for supporting T- and B-lymphocytes. DNase is essential for the mitigation of cell clumping as a result of DNA release from dying cells and post-mortem stress on deceased-donor derived BM. Heparin was used as an anticoagulant. HSA provided a protein source to prevent cell adherence and adsorption to surfaces (Fig. 1).

### Grinding and elution

An electric bone grinder was assembled in an ISO-5 cleanroom (Facility A), and a purpose-built bone grinder (Biorep Technologies Inc., Miami, FL) was assembled in a Biological Safety Cabinet (Facility B). In both facilities, a 2 L stainless steel beaker containing 100 mL of fresh processing medium was placed under the grinding head to catch bone fragments and media flow-through. Bone types were kept separate if both VB and IL from the same donor were processed. Processing medium was used to rinse the grinder throughout the process to prevent bone from drying and sticking to the chamber. Once all bone pieces were ground, the chamber was thoroughly rinsed with fresh processing media. The final volume in the stainless-steel beaker was typically around 750 mL.

Stainless steel sieves were stacked with a No. 40 (425 µm) on top of a No. 80 (177 µm) and seated over a round catch-pan (WS Tyler, St. Catherines, ON). The stainless-steel beaker was swirled and poured over the sieves. Bone fragments were distributed evenly on top of the sieve and rinsed with 250 mL of fresh processing medium. The sieved BM product, approximately 1000 mL, was transferred to a sterile pack for final analysis. The HSPC extraction process took 6–8 h following receipt by the processing facility, depending on the quality of the recovery and the number of VBs received.

### Nucleated cell counts

An aliquot of BM extract was subjected to red blood cell lysis with ammonium chloride RBC lysis buffer. In a 15 mL conical tube, 4 mL of 9% ammonium chloride was added to 1 mL of BM cell suspension and incubated for 5 min at room temperature. Following incubation, the lysed sample was filled to the top of the tube with IMDM containing 100 U/mL DNase, 10 U/mL heparin, and 2.5% HSA processing medium. The lysed sample was centrifuged at 300×*g* for 5 min and decanted. The sample was then washed with 15 mL of processing medium, centrifuged at 300 x g for 5 min, and decanted. Finally, the lysed cells were re-suspended with 1 mL of the same processing medium. Viable nucleated cell counts were obtained using Trypan blue and a hemocytometer.

### Flow cytometry

Flow Cytometry was performed using an ACEA Biosciences NovoCyte 2060R equipped with 488 nm and 640 nm lasers. ISHAGE methods were used to enumerate CD45+ and CD34+ cells [16]. 500 µL of lysed bone marrow extract was stained for 15 min with 2 µL each of CD45-FITC, CD34-APC, 7-AAD, and Annexin-PE. All conjugated antibodies were purchased from BD Biosciences and 7-AAD was obtained from Tonbo Biosciences. Cells were also stained with individual conjugate antibodies for controls and compensation. After incubating for 15 min, cells were washed with Dulbecco’s phosphate buffered saline, centrifuged, and re-suspended in 500 µL of PBS. These samples were run directly on the flow cytometer and analyzed using the ISHAGE gating scheme [16]. For each sample, 100,000 total events gated on the Singlets gate were collected.

### Colony forming unit (CFU) assay

The concentration of RBC lysed cell suspension was first adjusted to 105 viable cells/mL with processing medium before adding 250 µL to 2.5 mL of semisolid medium, Methocult Optimum (Stem Cell Technologies, Vancouver, Canada) and then vigorously vortexed to achieve adequate mixing. A 3 cc syringe was used to remove at least 2.2 mL of Methocult containing cells. 1.1 mL was dispensed into each of two 35 mm non-tissue culture treated dishes. The dishes were covered and tilted to ensure coating of the entire plate surface with Methocult. The two dishes were placed inside a larger 100 mm petri dish with a third uncovered 35 mm dish containing sterile deionized water to humidify the plate. Plates were incubated for 14 days at 37 °C, 5% CO_2_ before scoring colonies.

### Numbers of donors and bone marrow samples utilized for statistical modeling

Seventy-five bones from 62 donors were initially received by the two BM processing facilities. The numbers of samples with complete data records differed depending on the outcome being modeled. Table 1 provides a breakdown of the numbers received and the numbers with complete data available for statistical modeling by outcome.

**Table 1:** Numbers of donors and bones with complete data records available for analysis

Modeled Outcome	Donors	Bones	VB	IL
%CD34+	62	75	52	23
CFU-TOTAL/10^5^	54	67	42	25
CFU-GM 10^5^	54	66	41	25

### Definition of ischemia time

Total ischemia was defined as the time interval from death (when the donor’s arterial system was cross-clamped and circulation ceased) to start of BM recovery at the processing facility. For purposes of statistical modeling, this total interval was separated into three successive and mutually exclusive time components: (a) *Warm*-*Ischemia Time* (WIT): Beginning at time of death and ending either when bones were recovered and packed on ice, or when the body was placed in a cooler. (b) *Body*-*Cooling Time* (BCT): Beginning when the body was placed in the cooler and ending when recovered bones were packed on ice. (c) *Cold*-*Ischemia Time* (CIT): Beginning when recovered bones were packed on ice and ending when processing began for extraction of HSPCs. Thus, Total Ischemia Time = (WIT) + (BCT) + (CIT). When body cooling was not used, BCT was coded zero and Total Ischemia Time = (WIT) + (CIT). Ischemia times were considered the main variables of interest in statistical models.

### Definition of experience

Because this was the first series in our hands in which BM was processed from the bones of deceased donors, we hypothesized that HSPC quality might improve with learning as we gained more processing experience. This hypothesis rests on long-established research demonstrating that learning exerts significant effects on outcomes and costs in both industrial manufacturing [17] and medical practice settings [18–20]. To control for learning, we created a variable, EXPERIENCE, defined as the number of donors processed prior to the current one. For the ith donor, EXPERIENCE was coded i − 1, to indicate that EXPERIENCE is always one less than the serial number of the current case being processed. Because Facility A began processing BM 5 months before Facility B, and because Facility B had the advantage of participating in and learning from cases processed at Facility A, we hypothesized that the two facilities would have different learning trajectories. To account for this possible difference, each facility’s experience was coded separately. To identify the facilities in the model, we coded Facility A = 1 and Facility B = 0. The effect of EXPERIENCE was initially estimated in separate regression models and subsequently incorporated as a covariate in final adjusted models to control for the effect of learning on outcomes (see Additional file: Appendix S1 for details).

### Other covariates

Other variables tested in statistical models were: (1) BONE TYPE, vertebral bodies (VB) and ilia (IL), (representing the two sources of BM cells, coded VB = 1; IL = 0); DONOR SEX (percent male); and DONER AGE (years). These additional covariates were treated as exogenous factors and were included in final models only if they were statistically significant, or they improved the model’s performance.

### Outcome variables

Outcomes were defined according to three hallmarks of potential in vivo utility: (a) The proportion of recovered CD34+ cells that were viable (%CD34+) determined by 7-AAD, (b) The total number of colony forming units (CFUs) per 10^5^ total nucleated cells (TNC) plated (CFU-TOTAL), and (c) The number of CFU granulocyte-macrophages detected per 10^5^ nucleated cells (CFU-GM).

### Summary statistics

Donor and processing-facility characteristics, ischemia times, and outcome measures were summarized as means or percentages as appropriate. Crude (unadjusted) comparisons between FACILITIES (A vs. B), BONE TYPE (VB vs. IL), and BODY COOLING (Yes or No) were tested using independent-groups t-Tests or z-tests for proportions.

### Statistical modeling

Details of our general modeling approach are provided in the Additional file: Appendix S2. Briefly, the associations of ischemia times with outcomes were initially investigated in unadjusted regression models using only ischemia times as predictors. Additional models were then estimated to determine the separate associations of EXPERIENCE with outcomes. Finally, the effects of ischemia were evaluated in multivariable models, which controlled for the potential influences of FACILITY, EXPERIENCE, BONE TYPE, DONOR SEX, and DONOR AGE. Separate models were estimated for each of the three outcomes of interest (%CD34 + , CFU-TOTAL, and CFU-GM).

Ordinary least-squares (OLS) linear regression was initially employed to test a range of candidate models, including models incorporating two-way interactions, as well as logarithmic and second-order polynomial terms. From these candidates, the best reduced models were selected. Criteria used to select reduced models are described in Additional file: Appendix S2.

Because %CD34+ is a *proportion*, limited to the unit interval, [0 ≤ (%CD34+) ≤ 1], we found that traditional OLS linear regression produced unrealistic fitted values exceeding these interval boundaries. To correct for this, we substituted beta regression [21] for linear regression in models of %CD34+. Beta regression is useful in situations where the response variable is a rate or proportion measured on a continuous scale and bounded by minimum and maximum values. A technical description of beta regression is provided in Additional file: Appendix S2.

#### Model validation

Final models were validated using leave-one-out bootstrap cross-validation [22], which was accomplished by randomly omitting one observation with replacement from the dataset and re-estimating the model from the remaining observations. The resulting model was then used to predict the omitted observation. This procedure was repeated 200 times, yielding 200 models with predicted values, model coefficients, standard errors, and 95% confidence intervals. Model parameters were summarized as averages of the 200 bootstrapped models. Since bootstrap models are naïve to the omitted observations, this form of validation serves as an estimate of the predictive accuracy likely to be seen when the original model is used to predict new observations [23]. Model coefficients are reported for the original models and compared with averaged coefficients ± 95% confidence intervals from the 200 cross-validated models.

## Results

Sample characteristics are provided in Table 2.

**Table 2:** Sample characteristics. Numbers are those associated with the %CD34+ model

	Mean/percent	± Std error	Min	Max
Bone type (% vertebrae)	65.2%	5.32%	–	–
Donor sex (% male)	77.2%	0.54%	–	–
Donor age (years)	41.2	1.6	13	64
Experience^a^	26.9	1.2	0	53
Warm ischemia (h)	3.6	0.4	0.05	13.4
Body cooling (h)	7.9	0.9	0	22.5
Cold ischemia (h)	19.6	1.2	7.4	67.8
Total ischemia (h)	31.0	1.2	15.3	70.5
Outcomes				
%CD34+ viability (n = 75)	79.3%	3.0%	15.1%	100%
CFU-TOTAL/10^5^ cells (n = 67)	250.3	49.5	0	1850
CFU-GM/10^5^ cells (n = 66)	38.2	7.8	0	282

^a^Average number of cases processed prior to the current case

The statistical distributions of individual ischemia-time components WIT, CIT, and body cooling time (BCT) ordered by total ischemia time, for each of 62 donors is provided in the Additional file: Appendix S3, Figure S1.

### Unadjusted comparisons

Comparisons of FACILITY, BONE TYPE, and BODY COOLING are displayed in Additional file: Appendix S4, Table S4. The distributions of donor age and donor sex did not differ significantly by FACILITY, BONE TYPE, or whether BODY COOLING was used.

Processing facilities differed significantly in the distribution of BONE TYPE (27% of the bones processed at Facility A were VBs, while 100% of the bones processed at Facility B were VBs), which occurred because Facility B was structured to receive VBs only. Facility A also had more processing experience (Facility A = 53 bones processed vs. Facility B = 24 bones processed; p < 0.00001), longer WITs (Facility A = 3.55 h vs. Facility B = 2.13 h; p = 0.003), and shorter CITs (Facility A = 19.55 h vs. Facility B = 28.38 h; p = 0.004). The two facilities did not differ significantly in either BCT or total-ischemia time. Outcomes between the two facilities differed only for CFU-GM counts, with Facility A having significantly lower counts than Facility B (28.38 vs. 64.31, respectively; p = 0.04). No differences were detected in the percentage of viable CD34+ or CFU-TOTAL. Facility differences were controlled in final regression models.

Differences in BCT by BONE TYPE (middle section of Additional file: Appendix S4, Table S4) approached significance (p = 0.09), with the processing of VBs associated with shorter BCTs (6.32 h) compared to IL (8.51 h). This occurred because Facility B, which processed only VBs, had shorter BCTs than Facility A. Outcomes also differed by BONE TYPE. Compared to IL, VBs yielded higher numbers of CFU-TOTAL (341.29 vs. 97.44 per 10^5^ cells, respectively; p = 0.02) and CFU-GM (50.46 vs. 18.03 per 10^5^ cells, respectively; p = 0.04). BONE TYPE was controlled in final regression models.

In cases where the body was refrigerated prior to bone recovery (right most section of Additional file: Appendix S4, Table S4), mean WITs tended to be significantly shorter (2.65 h *with*, vs. 3.98 h *without* body cooling, p = 0.04). The same was true for CITs (19.51 h *with*, vs. 28.83 h *without* body cooling, p = 0.009). It is noteworthy that all outcomes were worse when body cooling was employed. Mean %CD34+ viability was 72.75% *with*, vs. 89.86% *without* body cooling (p = 0.0001). Similarly, with and without body cooling the average CFU-TOTAL count was 100.16 vs. 659.00 per 10^5^ TNC plated, respectively (p ≤ 0.00001), and the average CFU-GM count was 18.52 vs. 94.85 per 10^5^ TNC plated, respectively (p < 0.00001). Body cooling was accounted for in both initial and final ischemia-time regression models.

### Ischemia-time regression models

Unadjusted (base) regression models used only WIT, BCT, and CIT as predictors (no adjustments for other covariates). These models are summarized in the Additional file: Appendix S3, Tables S3 (a–c). Since BONE TYPE, FACILITY, and EXPERIENCE were found to be significant variables associated with outcomes (Additional file: Appendix S4, Table S4), adjusted models were developed to control statistically for the influence of these other covariates.

The adjusted beta regression model predicting %CD34+ viability is shown in Additional file: Appendix S4, Table S5. (Details of beta regression are provided in the Additional file: Appendix S2). The percentage of viable CD34+ cells that were recovered, declined significantly as a function of increasing BCT, with the decline occurring at a diminishing rate as %CD34+ approached zero (linear effect, p = 0.002; second-order polynomial effect, p = 0.03). A similar curvilinear decline in %CD34+ occurred in relation to increasing CIT (linear effect, p = 0.003; second-order polynomial effect, p = 0.005). Neither BONE TYPE nor WIT were significant. EXPERIENCE (p = 0.09) and the FACILITY x EXPERIENCE interaction (p = 0.07) approached statistical significance. Odds ratios measure the change in %CD34+ associated with a one-unit change in the associated predictor variable. For example, the odds ratio associated with a 1-h increase in WIT is 0.9663, indicating that each one-hour increase in WIT reduces %CD34+ to 96.63% of its previous value. The model’s predictive validity is evidenced by the similarity of the estimated parameters of the original model (left panel of Additional file: Appendix S4, Table S5) to the average of those of the bootstrap models (right panel). The model is statistically significant (p = 0.001).

Results of the adjusted linear regression of CFU-TOTAL is shown in Additional file: Appendix S4, Table S6. Here the importance of BONE TYPE as a source of BM cells is revealed. When BM cells were recovered from VB rather than IL, CFU-TOTAL increased by 207/10^5^ TNC plated (p = 0.025). The effect of BCT on CFU-TOTAL was negative. As BCT increased, the recovery of CFU-TOTAL decreased, with the decline occurring at a diminishing rate (linear effect, p = 0.00005; second-order polynomial effect, p = 0.002). The effects of WIT and CIT were not statistically significant. EXPERIENCE also was not significant; however, EXPERIENCE was retained because model performance improved when EXPERIENCE was controlled statistically. When BONE TYPE and EXPERIENCE were both controlled statistically, the model’s explanatory power improved from R^2^ = 35% to 47%. The adjusted R^2^ also improved from 35% to 40%, indicating that the improvement was not the result of model over-specification. Model precision also improved, as indicated by smaller standard errors. The similarity of the estimated parameters of the original model (left panel of Additional file: Appendix S4, Table S6) to the averaged results of the bootstrap models (right panel) is evidence of the model’s predictive validity. The model was significant (p = 0.000005) and explained 47.3% of the total variation in CFU-TOTAL.

Results of the adjusted linear regression of CFU-GM are shown in Additional file: Appendix S4, Table S7. The best CFU-GM model included BONE TYPE as a control variable, but not EXPERIENCE or FACILITY. Although BONE TYPE was not statistically significant, it was retained in the model because its inclusion improved the explanatory power from R^2^ = 32% to 34%, while the adjusted R^2^ remained the same (29%), suggesting the model was not over-specified. With BONE TYPE controlled statistically, WIT and BCT continue to demonstrate statistically significant associations with CFU-GM. Each passing hour of WIT reduces CFU-GM by − 7.19/10^5^ TNC plated (p = 0.03), while each hour of BCT reduces CFU-GM by − 5.24/10^5^ TNC plated (p = 0.00003). CIT had no effect (p = 0.86). The model’s predictive validity is evidenced by the similarity in the parameters of the original model (left panel of Additional file: Appendix S4, Table S7) to the averaged results of the bootstrap models (right panel). The model is significant (p < 0.00001) and explains one-third of the variability in CFU-GM.

### Predictions of cell viability from %CD34+ model

**Fig. 2:**
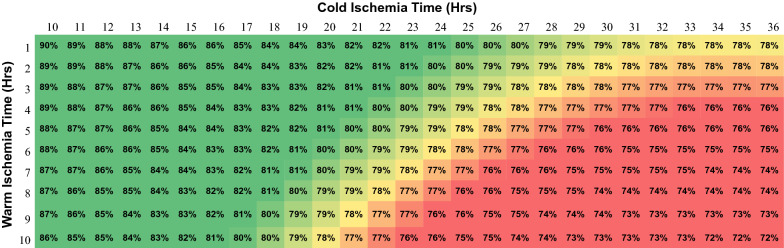
Predicted values from the %CD34+ beta regression model generated from the coefficients in Additional file: Appendix 4, Table S5. Calculated values in each square represent the percentage of viable CD34+ cells recovered from whole BM. The gradient of shading demonstrates the overall interrelationship between WIT and CIT. Green shading represents values above 80% viability, red shading below 80% and yellow near an 80% threshold. Input values used in the beta regression model to calculate CD34+ viability predictions were as follows: BCT = 0 h (no body cooling); Facility B = 0 (Indianapolis); Experience = 12 (mean for Indianapolis); Bone Type VB = 1 (vertebral bodies). WIT and CIT values are varied from the 10th to 90th percentile of observed values

The pattern of predictions shown in Fig. 2 illustrates how various combinations of WIT and CIT alter the viability of recovered CD34+ cells. These predictions were generated from the beta regression model of Additional file: Appendix S4, Table S5 with BCT set to zero (i.e., no body cooling). Green shading represents predicted CD34+ viabilities above 80%, red shading below 80%, and yellow near an 80% threshold. Figure 2 shows, as one would expect, that WIT is more detrimental to cell viability than CIT. When WIT is held to 3 h or less, the viability of CD34+ cells remains at or above 80% (green region) for up to 24 h of CIT. However, as WIT is extended beyond 3 h, the amount of CIT that can be tolerated is progressively shortened. We did not test the effect of cryopreservation; therefore, these predictions do not account for possible loss of viability due to subsequent freezing and thawing of recovered cells. The lesson from Fig. 2 is that longer total ischemia times can be tolerated when warm ischemia is kept to a minimum. Similar predictions for CFU-TOTAL and CFU-GM can be made using the coefficients provided in Additional file: Appendix S4, Tables S6 and S7.

## Discussion

In terms of total numbers of donors and number of bones procured, this pragmatic observational study is the largest to date, and the first to quantify the influences of ischemia and body-cooling times on the quality of HSPCs recovered from the bones of deceased donors under realistic field conditions. Because the study was designed to optimize externally valid inferences, i.e., inferences that are generalizable to usual practice settings, the study covers the full continuum of ischemia times likely to be seen under normal OPO operating conditions, and differs from previous studies conducted at single institutions, where donor bones were recovered immediately after cardiac arrest (i.e., no body cooling), with rapid bone recovery (i.e., short WIT), and without the need for long periods of transport (i.e., reduced CIT) [4, 14, 15].

The study results convey four principal messages. First, acceptable levels of HSPC quality are achievable despite the prolonged ischemia times that are inevitable when bones must be procured by geographically dispersed OPOs and shipped cross-country to a distant processing center. Our analyses show that under such conditions, favorable combinations of warm- and cold-ischemia times are achievable, enabling CD34+ cell viabilities in the range of 80–90%. Overall, the mean percentage of viable CD34+ cells recovered over all ischemia times was just under 80% (79.3%, Table 2).

The second message is that refrigerating the body prior to bone recovery, a practice that is common in the recovery of tissues, is detrimental to the viability and function of HSPCs recovered from deceased donor BM. Previous studies have demonstrated it takes over 10 h for a 37 °C, 70 kg body to cool to 4 °C when placed into a 4 °C morgue refrigerator [24]. This process is dependent upon body weight and is dynamic. The cooling curve becomes asymptotic as the body approaches the surrounding temperature and is much slower than cooling a smaller mass such as a block of VBs weighing ~ 3 kg on wet ice. While body cooling has been demonstrated to be appropriate for the recovery of non-viable tissues, in the context of bone marrow recovery either the duration of time at metabolically relevant temperatures, the longer cooling curve, or an interaction of both is apparently detrimental. When whole-body cooling was used, CD34+ cell viability averaged 72.75%; when body cooling was *not* used, average viability increased to nearly 90% (89.96%, Additional file: Appendix S4, Table S4), suggesting that the optimal practice would be to dispense with body cooling and transfer recovered bone as quickly as possible to a cold ischemic environment.

Third, our analyses suggest that the source of BM (bone type) matters. In unadjusted comparisons, CD34+ cell viabilities were > 80% when VBs were the source, vs. < 74% when IL were the source. Similarly, CFU-TOTAL and CFU-GM counts for VBs vs. IL were 341.29 vs. 97.44, and 50.46 vs. 18.03, respectively (Additional file: Appendix S4, Table S4). The reason for these differences is not entirely clear and is probably multifaceted. It is likely that variation in the recovery and isolation process used with the two bone types played a more important role than physiological differences. For example, VBs were exposed to fewer hours of body cooling on average than IL (6.32 vs. 8.51 h, respectively; Additional file: Appendix S4, Table S4), and body cooling, in turn, was associated with poorer outcomes. When the influence of body cooling was controlled in regression models, the differences in outcomes between VB and IL were reduced for CFU-TOTAL (Additional file: Appendix S4, Table S6), and were reduced and became statistically nonsignificant for CFU-GM (Additional file: Appendix S4, Table S7). Thus, the observation that VBs yield higher-quality HSPCs than IL is partially explained by the skewed distribution of body cooling between the two bone types. The influence of VB on %CD34+ was not statistically significant in unadjusted comparisons (Additional file: Appendix S4, Table S4), and remained nonsignificant after adjusting for body cooling (Additional file: Appendix S4, Table S5). Given that this is the first study to compare BM from deceased donor VBs and IL, no other directly comparable data exist. The closest approximation is comparisons of the viability of CD34+ cells recovered from the BM of deceased donor VBs and living donor aspirated iliac crest, which showed no difference [25–27].

The fourth message that our analysis conveys is that experience (learning) is a relevant factor that may vary substantially across different processing centers. As with most technical activities, the processing of BM cells from deceased donor bone follows a learning curve. It is well established that the products of industrial manufacturing improve with learning, a phenomenon first documented over 80 years ago [28] and subsequently incorporated into standard textbooks on operations management [17, 29]. In more recent times, it has been shown that the learning-curve phenomenon extends to both the outcomes and costs of medical procedures [18–20]. We observed different learning trajectories in the BM processing centers we studied, and, although we analyzed only two centers, our results suggest that the pace of learning and the shape of the learning curve may vary substantially across centers, a factor that should be considered in the design of future training programs, BM processing protocols, and certification practices. Our analysis implies that a volume-outcome relationship exists for the processing of BM and that a high-volume, regional center that has accumulated more processing experience may produce a higher-quality BM product compared to a low-volume center.

The intent of the present study was not to optimize yield or viability; however, some comparisons can be made to previous reports of deceased human-donor BM recovery, where optimization *was* the goal. Three previous studies have compared BM from a combined total of 99 deceased donors to that of a combined 58 living donors [25–27]. In these reports, the percentage of viable CD34+ cells from deceased donor BM (2.1 ± 0.6%; mean ± standard deviation) was not statistically different (p = 0.32) than BM aspirated from living donors (1.56 ± 0.92%). This compares well with our findings, in which the average percentage of CD34+ cells recovered was 2.43 ± 0.64% (mean ± SEM). We did observe greater variation in CD34+ percentages, which likely reflects the extreme range of ischemia times and, consequently, the quality of donor tissue on arrival in our study. Additionally, in this study the average total nucleated cell counts recovered from each donor was 9.1 × 10^7^ (range: 1.3 × 10^6^ to 6.4 × 10^8^) per gram of bone processed from an average bone weight of 312.4 ± 58 g (mean ± SEM). This would equate to CD34+ cell yields ranging from ~ 32,000 to > 155 M per donor. Again, this range reflects an extreme span of ischemia times as this study was intended to determine acceptable boundaries to carry forward into subsequent research. Previous studies under ideal conditions have reported TNC recoveries of 5.1 ± 1.1 × 10^10^ (mean ± SD) [4, 14, 15].

The quality of deceased-donor CD34+ HSPCs also has been compared to living-donor HSPCs by assaying CD34+ cell viability and CFU potential [25–27]. These studies reported a mean viability of 95.2 ± 3.6% for CD34+ cells recovered from deceased donors, compared to 93.5 ± 0.35% for living donors. Functional equivalency of deceased-donor and living-donor BM HSPCs, established by comparing the frequency of CFU-GM, was 105 ± 65 per 10^5^ TNC plated in deceased-donor BM, compared to 81.4 ± 17 per 10^5^ TNC plated in living donor BM. By comparison, our overall averages from deceased-donor BM (Table 2 and Additional file: Appendix S4, Table S4) were lower for both of these quality metrics, presumably due, again, to the extreme range of ischemia times and the inclusion of body cooling in our study, which negatively impacted average cell functionality. We have subsequently used the findings reported here to establish limits of 8 h WIT and 40 h CIT, and have eliminated the practice of body cooling. Following this protocol change, vertebrae from 50 donors meeting these restrictions have now been recovered and processed (manuscript in preparation). The average CD34+ HSPC viability was 90.5 ± 1.9% and the average CFU-GM was 158.3 ± 13.5/10^5^ TNC plated, which is comparable to the previous studies [25–27].

Overall, our study demonstrates the feasibility of recovering viable BM from deceased donors for banking. Building a BM bank with sufficient HLA diversity requires an ample source and steady supply of deceased-donor medullary bones. Fortunately, the Uniform Anatomical Gift Act of 1968 established a syndicate of 58 geographically distributed OPOs. Each year approximately 10,000 deceased individuals donate their organs, and a further 40,000 donate tissues, yielding approximately 30,000 organs and over a million tissues recovered annually (unos.org/data/transplant-trends/accessed 16 March 2020). The high volume of bones potentially recoverable through this network, could provide the necessary inventory to justify the establishment of an integrated system of bone procurement, recovery, and transport, linked to BM processing and banking centers. An integrated system of this type would require the cooperation and coordination of multiple OPOs all following agreed-upon operational protocols. Our study demonstrates the feasibility of building such a system using existing OPO infrastructure. In particular, we have demonstrated that protocols designed to maintain a favorable ischemic environment from the point of bone procurement and recovery, through cross-country shipping, to arrival at the BM processing center, can be developed and enforced. Because our data encompassed the full range of body-cooling and ischemia times likely to be seen in practice, they possess a high level of external validity (generalizability), and our predictive models (Fig. 2) can be used to establish realistic ischemia-time tolerance limits for satisfying HSPC quality-acceptance standards.

The creation of a BM banking system would offer several distinct advantages over current living-donor registries. First, personal risk to live donors would be obviated, as opposed to only ameliorated through the present predominant practice of mobilized peripheral blood collection. Second, much larger volumes of HSPCs can be recovered from a deceased donor compared to a living donor, allowing for multiple or repeat infusions from the same donor in cases of graft failure. Additionally, recovered cells can be packaged in known quantities, tested for quality, and cryopreserved for later on-demand use. Because the units are cryopreserved, they can be stored indefinitely [30], thereby obviating the problem of attrition that occurs with living-donor registries. Finally, a BM bank can serve as a readily available resource during surges in demand following a mass casualty event, such as a nuclear disaster resulting in widespread bone-marrow failure [1, 2].

## Conclusions

Deceased-donor BM banking is now coming into existence and is beginning to display significant potential. From the perspective of a nascent processing facility, the results of our statistical models can be used to establish quantitative ischemia tolerance limits and quality acceptance standards for safeguarding the viability and function of HSPCs derived from deceased-donor bone. From a broader policy perspective, our models can also provide the foundation for an emerging BM banking system to institute data-driven industry standards.

## Supplementary information

**Additional file: Supplemental files detailing the statistical modeling and approach. Appendix S1. Experience Models, Appendix S2. General Modeling Approach, Appendix S3. Unadjusted (Base) Ischemia-Time Regression Models, Appendix S4. Final Adjusted Regression Models.**

## Data Availability

All data generated or analyzed during this study are included in this published article and its additional information files. Any dataset used and/or analyzed during the current study are available from the corresponding author on reasonable request.
