# Supplementary material for: Ischemia considerations for the development of an organ and tissue donor derived bone marrow bank
Source: J Transl Med. 2020 Aug 5;18:300. doi: 10.1186/s12967-020-02470-1 (PMC7405448; doi:10.1186/s12967-020-02470-1)
Supplement: Supplementary file 1 — Additional file: Supplemental files detailing the statistical modeling and approach. Appendix S1. Experience Models, Appendix S2. General Modeling Approach, Appendix S3. Unadjusted (Base) Ischemia-Time Regression Models, Appendix S4. Final Adjusted Regression Models. [file 12967_2020_2470_MOESM1_ESM.docx]

Appendix S1

Experience Models

To account for learning, we created a variable, EXPERIENCE, defined as the number of donors processed prior to the current donor. Donors were numbered serially from i = 1 . . . n, in the order they were processed, and EXPERIENCE was coded i – 1, to denote the fact that EXPERIENCE was always one less than the current donor being processed. Since Facility A began processing bone marrow five months before Facility B, and because Facility B had the advantage of participating in (and learning from) cases processed at Facility A, the two facilities had different learning trajectories. To account for this difference, EXPERIENCE was coded separately for each facility. To identify the two facilities in the model, we coded FACILITY A = 1 and FACILITY B = 0.

Experience models:

Outcomes (%CD34+, CFU-TOTAL/10^5^, and GM-TOTAL/10^5^) were modeled as linear combinations of FACILITY (where the processing occurred), EXPERIENCE (number of cases processed at the facility prior to the current case), and the FACILITY x EXPERIENCE interaction. %CD34+ was modeled using beta regression (see Technical Appendix B). The other two outcomes (CFU-TOTAL and CFU-GM) were modeled using traditional ordinary least-squares (OLS) linear regression. Models had the following general linear form:

[A.1] Y = β_0_ + β_1_(FACILITY) + β_2_(EXPERIENCE) + β_3_(FACILITY x EXPERIENCE)

Where: Y = Outcome (%CD34+, CFU/10^5^, or GM/10^5^)

β_0_ = Intercept (constant term)

β_1_ = Coefficient associated with FACILITY

β_2_ = Coefficient associated with EXPERIENCE

β_3_ = Coefficient associated with the FACILITY x EXPERIENCE interaction

The interaction term, β_3_, accounts for the possibility that Facility A may have a different linear relationship with EXPERIENCE (a different learning trajectory) than Facility B.

Algebra for deriving testable effects

The model for Facility A is:

[A.2] Y = β_0_ + β_1_(FACILITY A) + β_2_(EXPERIENCE) + β_3_(FACILITY A x EXPERIENCE)

= β_0_ + β_1_(1) + β_2_(EXPERIENCE) + β_3_(1 x EXPERIENCE)

= (β_0_ + β_1_) + (β_2_ + β_3_) x (EXPERIENCE)

The model for Facility B is reduced because Facility B = 0 and, therefore, the terms associated with β_1_ and β_3_ drop out of the model. Thus, the model for Facility B is:

[A.3] Y = β_0_ + β_1_(FACILITY B) + β_2_(EXPERIENCE) + β_3_(FACILITY B x EXPERIENCE)

= β_0_ + β_1_(0) + β_2_(EXPERIENCE) + β_3_(0 x EXPERIENCE)

= β_0_ + β_1_(0) + β_2_(EXPERIENCE) + β_3_(0)

= (β_0_) + (β_2_) x (EXPERIENCE)

The difference between Equations [A.2] and [A.3] provides insights into the effects that are testable in the model:

[A.4] FACILITY A: Y = (β0 + β1) + (β2 + β3) x (EXPERIENCE)

− FACILITY B: Y = (β0) + (β2) x (EXPERIENCE)

____________________________________________________

FACILITY A − B: (β1) + (β3) x (EXPERIENCE)

From [A.4], the following effects are testable as null hypotheses:

H0_1_: β_0_=0 Tests the significance of FACILITY B’s intercept relative to an intercept of zero. (The outcome for Facility B when EXPERIENCE=0).

H0_2_: β_1_=0 Tests the significance of FACILITY A’s intercept relative to FACILITY B’s intercept. (The outcome for FACILITY A relative to FACILITY B when FACILITY A’s EXPERIENCE=0).

H0_3_: β_2_=0 Tests the significance of the learning slope for Facility B (the change in FACILITY B’s outcome associated with each additional learning EXPERIENCE).

H0_4_: β_3_=0 Tests the significance of the learning slope for FACILITY A relative to FACILITY B’s slope.

Example calculation

Following is a worked example for predicting the effect of EXPERIENCE on CFU-GM/10^5^ given the regression coefficients and observed data values in Table S.1:

**Table S1.** Regression coefficients and ischemia time values

| Linear Regression  Coefficients | Predictor Variable | Observed Data |
| --- | --- | --- |
| β_0_ = 111.91 | Constant |  |
| β_1_ = -99.34 | FACILITY | A=1; B=0 |
| β_2_ = -3.57 | EXPERIENCE | 20 Previous Cases |
| β_3_ = 4.17 | FACILITY x EXPERIENCE | A=(1 x 20); B=(0 x 20) |

Assume that both FACILITY A and FACILITY B have processed 20 previous donors, what would their respective outcomes be for the 21^st^ donor?

[A.5] Expected outcome for FACILITY B:

CFU-GM/10^5^ = (β_0_) + (β_2_) x (EXPERIENCE)

= 111.91 + (-3.57) x (20)

= 40.51

Interpretation for FACILITY B:

For FACILITY B, starting with no experience (EXPERIENCE = 0), the beginning CFU-GM yield (intercept term) is expected to be β_0_ = 111.91 CFU-GM/10^5^. Each additional donor processed is then expected to subtract β_2_ = −3.57 CFU-GM/10^5^ from FACILITY B’s beginning amount. For the 21st donor processed, having learned from processing 20 previous donors, FACILITY B’s expected yield would be (β_0_) + (β_2_ x 20) = 111.91 + (−3.57 x 20) = 40.51 CFU-GM/10^5^.

[A.6] Expected outcome for FACILITY A:

CFU-GM/10^5^ = (β0 + β1) x (FACILITY) + (β2+ β3) x (FACILITY x EXPERIENCE)

= (111.91− 99.34) x (1) + (−3.57+4.17) x (1 x 20)

= (12.57) + (0.60 x 20)

= (12.57) + (12.00)

= 24.62

Interpretation for FACILITY A:

FACILITY A’s yield with no experience is estimated to be β_0_ + β_1_ = [111.91 + (– 99.34)] = 12.57 CFU-GM/10^5^ units more than FACILITY B’s. Each additional case that FACILITY A processes adds β_2_ + β_3_ = (−3.57 + 4.17) = 0.60 CFU-GM/10^5^ to FACILITY A’s starting yield. For the 21st case processed, FACILITY A’s yield would be (β_0_ + β_1_) + (β_2_ + β_3_) x (20) = 12.57 + (0.60 x 20) = 24.57 CFU-GM/10^5^. Note that Facility A’s learning slope is positive, adding β_3_ = 4.17 CFU-GM/10^5^ for each additional case processed, while Facility B’s learning slope is negative, subtracting β_2_ = −3.57 CFU-GM/10^5^ for each case. This is illustrative of a classic interaction. Expressed in relative terms, each additional learning experience is associated with a net gain of β_2_ + β_3_ = (−3.57 + 4.17) = 0.60 CFU-GM/10^5^ for FACILITY A relative to Facility B.

These examples illustrate the pattern that emerged for all three outcomes. FACILITY A, which began processing BM cells before FACILITY B, started at a relatively lower performance level and improved monotonically with each additional case processed. By comparison, FACILITY B, having participated in (and learned from) FACILITY A’s initial work, started at a higher level of performance but did not change significantly or declined slightly with increasing experience.

Appendix S2

General Modeling Approach

Linear Regression:

Ordinary least-squares (OLS) linear regression was initially employed to test a range of candidate models, including models incorporating two-way interactions, as well as logarithmic and second-order polynomial terms. From these candidates, the best reduced models were selected based on the following criteria. (a) Models with the greatest explanatory power (highest R^2^ values) were favored. (b) Parsimonious models that explained the greatest percentage of variation with the fewest predictors were favored. The *adjusted* R^2^, which guards against over-specification by penalizing models containing greater numbers of predictors (21), was used as a comparative indicator of explanatory power in selecting the most parsimonious models. Models that achieved the highest R^2^ values, while simultaneously maintaining or increasing the adjusted R^2^, were favored. (c) Models with greater precision, as indicated by relatively smaller standard errors associated with both the model and model coefficients were favored. (d) Models with the best fit, as judged by an assessment of residual plots, were favored. Residuals were plotted and examined visually for discernable patterns, and confirmed quantitatively by regressing residuals onto observed values to uncover possible interactions or underlying curvilinear relationships.

Beta Regression:

Because it is a ratio, *%CD*34*+* is confined to the closed unit interval (0 ≤ %CD34+ ≤ 1), meaning that it can assume values of 0% or 100% or any value in between, but it cannot be less than 0% or greater than 100%. Given this range restriction, we found that OLS linear regression produced unrealistic fitted values that exceeded the interval boundaries—some of the predicted values were less than 0% and some exceeded 100%. To correct for this, we considered beta regression (1) instead of OLS linear regression for models of *%CD*34+. Maximum likelihood beta regression is used to model beta-distributed random variables, which makes it particularly useful in situations such as ours where the response variable is a rate or proportion measured on a continuous scale and bounded by minimum and maximum values. We continued to use OLS linear regression to model the other two outcome variables, CFU-TOTAL and CFU-GM.

In the main text, *CD*34*+* is used to denote the count of recovered CD34+ cells and *%CD*34*+* denotes the percentage of total CD34+ cells that were viable. That is:

$${\%CD34+ =(Viable CD34+)}/{[\left( Viable CD34+ \right)+(Nonviable CD34+)]}$$

Here we use *pCD*34 = *%CD*34*+* to denote the percent of recovered CD34+ cells that were viable. To ensure that the outcome was evaluable as a beta-distributed variable, we transformed *pCD34* as follows:

*pCD34**$= {[1+100\left( pCD34 \right)]}/{102}$

This transformation restricts *pCD34** to the open interval, (0 < *pCD34** < 1), thereby satisfying the distributional assumption that the outcome variable can approach but cannot *equal* 0% or 100%. The restricted proportion, *pCD34** was then modeled by beta regression. For ease of interpretation, predicted values from beta regressions were back transformed to obtain:

Pred(*pCD34*) = [(102(Pred(*pCD34**)) – 1) ∕ 100] = Pred(pCD34) = Pred(%CD34+).

The beta regression equation:

The beta regression equation utilizes the logit link function of the outcome to *η*, a linear predictor. Our basic beta regression equation for predicting pCD34* was:

$$\left[ B.1 \right] \eta={\ln[{pCD34}^{*}}/{(1 - {pCD34}^{*})]= \beta_{0}+ \beta_{1}(WIT)+ \beta_{2}(BCT)+\beta_{3}({BCT}^{2})}\beta_{4}(CIT)+\beta_{5}({CIT}^{2})$$

Where:

β_0_ = Constant (intercept)

β_1_ = Coefficient associated with warm ischemia time (WIT)

β_2_ = Coefficient associated with body cooling time (BCT)

β_3_ = Coefficient associated with body cooling time squared (BCT^2^)

β_4_ = Coefficient associated with cold ischemia time (CIT)

β_5_ = Coefficient associated with cold ischemia time squared (CIT^2^)

Example calculation:

To illustrate the calculations, we use the coefficients and ischemia times shown in Table S.2.

**Table S2.** Regression coefficients and ischemia time values

| Beta Regression  Coefficients | Predictor Variable | Observed Data  (Ischemia Times) |
| --- | --- | --- |
| β_0_ = 3.500 | Constant |  |
| β_1_ = -0.01996 | Warm Ischemia Time (WIT) | 1.92 WIT hours |
| β_2_ = -0.181 | Body Cooling Time (BCT) | 0.00 BCT hours |
| β_3_ = 0.007 | Body Cooling Time squared (BCT)^2^ | 0.00 BCT hours^2^ |
| β_4_ = -0.111 | Cold Ischemia Time (CIT) | 14.92 CIT hours |
| β_5_ = 0.002 | Cold Ischemia Time squared (CIT)^2^ | 222.606 hours^2^ |

We apply Equation [B.1] to solve for *η* as illustrated in [B.2], below:

$$\left[ B.2 \right] \eta={\ln[{pCD34}^{*}}/{(1 - {pCD34}^{*})]= \beta_{0}+\beta_{1}(WIT)+\beta_{2}(BCT)+\beta_{3}({BCT}^{2})+}\beta_{4}(CIT)+\beta_{5}({CIT}^{2})$$

= *3.500 + (-0.01996)(1.92) + (−0.181)(0) + (0.007)(0) + (-0.111)(14.92) + (0.002)(222.606)*

= 2.2507688

Because they are related to the outcome variable through a nonlinear function, the coefficients of the linear predictor, *η*, lack a simple intuitive meaning. However, by applying the *inverse link function* to *η* we obtain a result that is easier to interpret.

The inverse link function:

The inverse link function, ${\exp\left( \eta\right)}/{[1+exp}(\eta)]$, converts the linear predictor, *η*, to the expected value of the outcome variable ${\text{pCD}34}^{*}$:

$${E[pCD34}^{*}]= \frac{\exp(\eta)}{[1+\exp\left( \eta\right)]} = \frac{\exp[ \beta_{0}+\beta_{1}(WIT)+ \beta_{2}(BCT)+\beta_{3}\left( {BCT}^{2} \right)+\beta_{4}(CIT)+\beta_{5}({CIT}^{2})]}{1+\exp[\beta_{0}+\beta_{1}(WIT)+ \beta_{2}(BCT)+\beta_{3}\left( {BCT}^{2} \right)+\beta_{4}(CIT)+\beta_{5}({CIT}^{2})]}$$

Applying the inverse link function to the predicted value, *η* = 2.2507688, calculated in Equation [B.2], we obtain the expected value, E[pCD34*]:

$$\left[ B.3 \right] E[{\mathrm{pCD}34}^{*}] = \text{exp}(2.2507688) / [1 + \text{exp}(2.2507688] = 0.905 \approx90.5\%$$

This result is interpretable as the expected value of ${\text{pCD}34}^{*}$ for the specified values of the predictors given in Table S.2.

To interpret the result of [B.3] in terms of pCD34 (the percentage of viable CD34+ cells), we use the back transformation:

$${\text{[B.4] pCD}34}^{*}= \frac{1+100 \text{pCD}34}{102} \to\text{pCD}34=\frac{{\text{102 pCD}34}^{*}-1}{100}= \frac{[(102)(0.905)-1]}{100} =0.9131$$

Equation [B.4] says that for the values specified in Table S.2, the expected percentage of viable CD34+ cells is pCD34 = %CD34+ = 91.31%.

Using the beta regression equation to determine the expected impact on ${\text{pCD}34}^{*}$ of a one-unit change in a given predictor variable:

The beta regression coefficient for any given predictor can be used to estimate the impact on ${\text{pCD}34}^{*}$ of a one-unit change in that predictor, *controlling for all other predictors in the equation*. This is accomplished via exponentiation of the particular regression coefficient under consideration. For example, to calculate the impact of a one-hour increase in warm ischemia time (WIT) on the ratio of the percent of viable CD34+ cells to the percent nonviable CD34+ cells, the ratio under consideration is:

$$\frac{{pCD34}^{*}}{{(1-pCD34}^{*})}=\frac{exp(\eta)}{1+exp(\eta)}/\left[ 1-\frac{\exp\left( \eta\right)}{1+\exp\left( \eta\right)} \right]=exp(\eta)$$

From Table S.2, the regression coefficient associated with WIT is β_1_ = -0.01996. Therefore, the impact of a one-hour increase in WIT on the ratio of percent viable to percent nonviable CD34+ cells is:

$$\left[ B.5 \right] \frac{\text{exp}\left[ \beta_{0}+\beta_{1}\left( WIT+1 \right)+\beta_{2}\left( BCT \right)+\beta_{3}\left( {BCT}^{2} \right)+ \beta_{4}\left( CIT \right)+\beta_{5}\left( {CIT}^{2} \right) \right]}{\text{exp}\left[ \beta_{0}+\beta_{1}\left( WIT \right)+\beta_{2}\left( BCT \right)+\beta_{3}\left( {BCT}^{2} \right)+\beta_{4}\left( CIT \right)+\beta_{5}\left( {CIT}^{2} \right) \right]}=\exp\left( -0.01996 \right)=0.98$$

Equation [B.5] says that if WIT were increased by one hour, while the other variables in the equation (BCT and CIT) remained unchanged, the ratio of the percent viable to percent nonviable CD34+ cells would decrease by 2%, or 98% of its previous value. We previously calculated ${\text{pCD}34}^{*}$ = 0.905, in Equation [B.3]. Thus, a one-hour increase in WIT would reduce ${\text{pCD}34}^{*}$ to 0.98 x 0.903 = 0.885, a 2% reduction. Equivalently, this would reduce the predicted pCD34 from 0.913 to 0.893, approximately a 2% reduction. The multiplicative factor, 0.98, is a constant applicable to any unit change along the continuous range of warm ischemia times. Factors for the other predictors can be obtained in the same way to estimate the impact of a unit change in BCT and CIT on pCD34*.

Appendix S3

Unadjusted (Base) Ischemia-Time Regression Models

The statistical distributions of individual ischemia-time components WIT, CIT, and BCT, for each of 62 donors is shown in the bar chart in Figure S.1. The chart shows the relationships of WIT, CIT and BCT when the data are ordered from shortest to longest total ischemia times.

**Figure S1**. Cumulative ischemia times for all donors used in the study. Donors are ranked from shortest to longest total ischemia times, which is a composite of WIT, CIT, and BCT. Only donors for which complete data were available were considered.


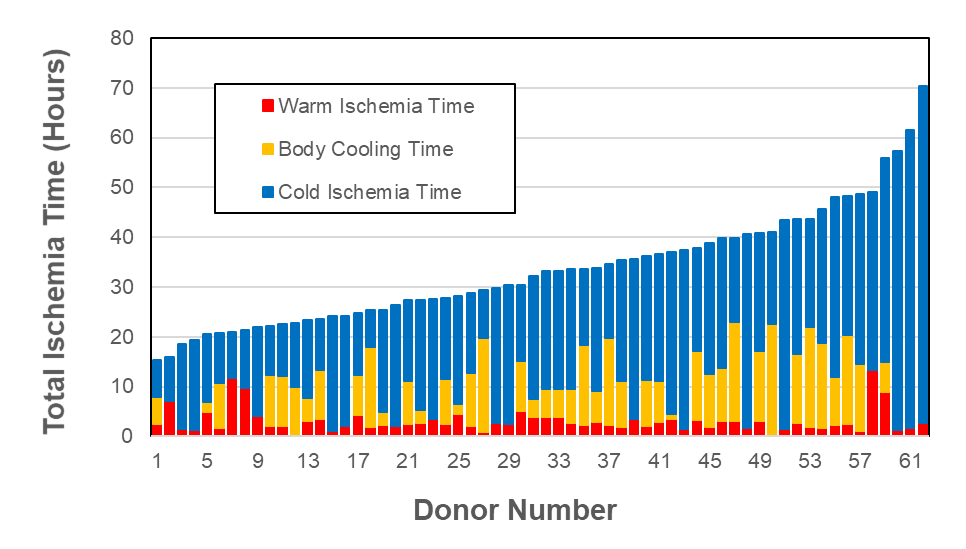


Initial (base) regression models used only WIT, BCT, and CIT as predictors of HSPC outcome (no adjustments for other covariates). Results of these models for %CD34+, CFU-TOTAL, and CFU-GM are shown in Table S.3(a-c). Model results are shown in the left panels of Table S.3(a-c); averaged results of 200 cross-validated models (each estimated with one observation omitted from the full dataset) are shown in the right panels.

Beta regression models for %CD34+ are shown in Table S.3(a). The relationship of WIT to %CD34+ was not statistically significant; however, BCT (linear component, p=0.001 and second-order polynomial component, p=0.01), and CIT (linear component, p=0.001 and second-order polynomial component, p=0.004) were both curvilinearly related to %CD34+. In both cases the CD34+ yield declines in response to increasing BCT and CIT, but then slightly increases at the upper extremes of BCT and CIT. The odds ratios in Table S.3(a) are continuous-variable ratios of viable CD34+ cells to total CD34+ cells and depict the impact of a one-unit change in a given predictor. These quantities are obtained via exponentiation of the regression coefficient associated with the particular predictor under consideration. For example, the coefficient associated with WIT is β_1_ = −0.01996. (Table S.3(a)) Exponentiation produces the following result:

e^β^ = e^-0.01996^ = 0.9802 or 98.02%,

which is the value of the odds ratio for warm ischemia shown in Table S.3(a). This result says that with BCT and CIT held constant, each one unit (one hour) increase in WIT reduces the ratio of viable to nonviable CD34+ to 98.02% of its previous value. The multiplicative factor, 0.9802, is a constant applicable to a one-unit change anywhere along the continuous range of WIT. Factors for the other predictors are provided in Table S.3(a) and can be used to estimate the effect of a one-unit change in BCT or CIT holding other variables in the equation constant. The beta-regression prediction equation is statistically significant (p = 0.0009).

The right half of Table S.3(a) shows the averaged results of bootstrapped cross-validations, which provide estimates of the original model’s validity in predicting future observations (2). If the original model was misspecified, the parameters of the re-estimated bootstrapped models would differ from the parameters of the original model. However, as revealed in Table S.3(a), model parameters (regression coefficients, standard errors, and 95% confidence intervals) associated with the original model (left panel) are nearly the same as the corresponding parameters generated through bootstrap re-sampling (right panel), providing evidence of the original model’s predictive validity when applied to future data drawn from the same population. [Technical details regarding beta-regression and example calculations are provided in Technical Appendix B, above].

Table S.3(b) shows linear regression results for CFU-TOTAL. The coefficients in linear regressions are direct estimates of the impact of a unit change in the associated predictor. BCT is the only statistically significant predictor in Table S.3(b). WIT and CIT are not significant. The relationship of BCT to CFU-TOTAL is curvilinear, indicating that each one-hour increase in BCT *decreases* CFU-TOTAL by −95.03639/10^5^ cells (p < 0.0001) while simultaneously *increasing* CFU-TOTAL^2^ by 3.45603/10^5^ (p = 0.0008). Together, the linear and second-order polynomial components combine to produce a decreasing trend in CFU-TOTAL that decays at a decelerating rate. The model parameters (left panel of Table S.3(b)) are similar to the averaged parameters of the bootstrapped models (right panel), providing evidence of the original model’s predictive validity. The model is statistically significant (p = 0.00002) and explains 35% of the variance in CFU-TOTAL.

Table S.3(c) shows linear regression results for CFU-GM. In this model the influences of WIT and BCT are statistically significant, while the influence of CIT is not significant. With CIT and BCT held constant, each hour of WIT reduces CFU-GM by -8.11295/10^5^ (p = 0.01). With WIT and CIT constant, each hour of BCT reduces CFU-GM by -5.52927/10^5^ (p < 0.000009). The right side of Table S.3(c) shows that the estimated parameters of the bootstrapped models are similar to those of the original model, again providing evidence of the original model’s predictive validity when applied to future data. The model is statistically significant (p=0.00002) and accounts for 32% of the total variation in CFU-GM counts.

**Table S3.** Base ischemia-time models

1. %CD34+ Beta Regression: Effects of warm ischemia, body cooling, and cold ischemia on number of viable CD34+ cells as a percentage of total CD34+

|  | Beta Regression Model | | | |  | Leave-One-Out Bootstrap Cross-Validation | | | | | |
| --- | --- | --- | --- | --- | --- | --- | --- | --- | --- | --- | --- |
| Predictor | Coefficient | Standard Error | p | Odds Ratio |  | Coefficient | Standard Error | Z | p | 95% C.I. | |
| β_0_ Constant | 3.5001 | 0.59 | <0.0001 |  |  | 3.5455 | 0.72 | 4.94 | <0.0001 | 2.14 | 4.95 |
| β _1_ Warm Ischemia (hrs)^a^ | -0.01996 | 0.05 | 0.71 | 0.9802 |  | -0.02085 | 0.06 | -0.37 | 0.709 | -0.130 | 0.089 |
| β _2_ Body Cooling (hrs) | -0.18145 | 0.05 | 0.001 | 0.8341 |  | -0.18498 | 0.06 | -3.21 | 0.001 | -0.298 | -0.072 |
| β _3_ Body Cooling Squared | 0.00664 | 0.003 | 0.01 | 1.0007 |  | 0.00680 | 0.003 | 2.26 | 0.02 | 0.0009 | 0.013 |
| β _4_ Cold Ischemia (hrs) | -0.11148 | 0.03 | 0.001 | 0.8945 |  | -0.11431 | 0.05 | -2.27 | 0.02 | -0.213 | -0.016 |
| β _5_ Cold Ischemia Squared | 0.00148 | 0.0005 | 0.004 | 1.0015 |  | 0.00106 | 0.001 | 1.44 | 0.15 | 0.0006 | 0.0036 |

Likelihood Ratio Chi-Square_(5)_ = 20.81, p = 0.0009, AIC = -80.68

^a^ Interpretation: For warm ischemia, the odds ratio is obtained from the regression coefficient as e^β^ = e^-0.019^ = 0.98. The odds ratio indicates that each one-hour increase in warm ischemia reduces the average percentage of viable CD34+ cells by 2% to 98% of its previous value. For example, with body cooling and cold ischemia held constant, a one-hour increase in warm ischemia would reduce the percentage of viable CD34+ cells by −1.3%, from an expected value of 67.6% to 66.3% (i.e., 0.98 x 67.6% = 66.3%). Odds ratios for the other predictors are obtained in the same way and have the same interpretation.

1. CFU-Total Linear Regression: Effects of warm ischemia, body cooling, and cold ischemia on number of CFUs per 10^5^ Cells

|  | Linear Regression Model | | |  | Leave-One-Out Bootstrap Cross-Validation | | | | | |
| --- | --- | --- | --- | --- | --- | --- | --- | --- | --- | --- |
| Predictor | Coefficient | Standard Error | p |  | Coefficient | Standard Error | Z | p | 95% C.I. | |
| β_0_ Constant | 756.5084 | 169.20 | <0.0001 |  | 713.8019 | 150.49 | 4.74 | <0.0001 | 418.845 | 1008.758 |
| β_1_ Warm Ischemia (hrs)^a^ | -9.10826 | 19.98 | 0.65 |  | -3.67879 | 30.92 | -0.12 | 0.90 | -64.278 | 56.921 |
| β_2_ Body Cooling (hrs) | -95.03639 | 20.02 | <0.0001 |  | -92.98267 | 23.58 | -3.94 | <0.0001 | -139.204 | -46.761 |
| β_3_ Body Cooling Squared | 3.45603 | 0.98 | 0.0008 |  | 3.39958 | 1.12 | 3.04 | 0.002 | 1.210 | 5.589 |
| β_4_  Cold Ischemia (hrs) | -4.53349 | 3.99 | 0.26 |  | -3.81438 | 3.82 | -1.00 | 0.32 | -11.306 | 3.678 |

Model F_(4,62)_ = 8.28, p = 0.00002, R^2^ =0.348, R^2^adj = 0.306

^a^ Interpretation: The linear regression coefficient associated with warm ischemia indicates that each one-hour increase in warm ischemia reduces the total number of CFUs by -9.11/10^5^ cells. With body cooling and cold ischemia held constant, a one-hour increase in warm ischemia from would reduce the expected CFU-TOTAL count from 93/10^5^ to 83.89/10^5^ (i.e., 93 – 9.11 = 83.89).

1. CFU-GM Linear Regression: Effects of warm ischemia, body cooling, and cold ischemia on number of CFUs per 10^5^ GM cells

|  | Linear Regression Model | | |  | Leave-One-Out Bootstrap Cross-Validation | | | | | |
| --- | --- | --- | --- | --- | --- | --- | --- | --- | --- | --- |
| Predictor | Coefficient | Standard Error | p |  | Coefficient | Standard Error | Z | p | 95% C.I. | |
| β_0_ Constant | 104.1805 | 24.66 | <0.00001 |  | 104.2596 | 26.40 | 3.96 | <0.0001 | -1.027 | 1.181 |
| β_1_ Warm Ischemia (hrs) | -8.11295 | 3.16 | 0.01 |  | -8.10758 | 2.73 | -2.97 | 0.003 | -13.466 | -2.759 |
| β_2_ Body Cooling (hrs) | -5.52927 | 1.14 | <0.00001 |  | -5.54669 | 1.17 | -4.74 | <0.0001 | -7.857 | -2.260 |
| β_3_ Cold Ischemia (hrs) | 0.08872 | 0.61 | 0.88 |  | 0.08278 | 0.56 | 0.14 | 0.89 | -1.027 | 1.180 |

Model F_(3,62)_ = 9.82, p = 0.00002, R^2^ =0.322, R^2^adj = 0.289

Appendix S4

Final Adjusted Regression Models

**Table S4.** Unadjusted comparisons of (a) Processing Facility, (b) Bone Type, and (c) Body Cooling used in the final adjusted regression models.

|  | Facility | | |  | Bone Type | | |  | Body Cooled (Y/N) | | |
| --- | --- | --- | --- | --- | --- | --- | --- | --- | --- | --- | --- |
|  | A | B | p |  | VB | IL | p |  | Yes | No | p |
| Bone Type (%VB) | 27.0% | 100% | ─ ^a^ |  | ─ | ─ | ─ |  | 64.7% | 79.2% | 0.18 |
| Donor Sex (% Male) | 78.7% | 73.7% | 0.67 |  | 74.4% | 82.6% | 0.44 |  | 77.8% | 77.8% | 0.95 |
| Donor Age (yrs) | 41.18 | 42.44 | 0.65 |  | 41.0 | 42.9 | 0.52 |  | 40.78 | 43.33 | 0.32 |
| Experience | 26.86 | 12.00 | <0.0000 |  | 20.9 | 24.1 | 0.45 |  | 23.74 | 18.0 | 0.12 |
| Warm Ischemia (hrs) | 3.55 | 2.13 | 0.003 |  | 2.90 | 3.47 | 0.38 |  | 2.65 | 3.98 | 0.04 |
| Body Cooling (hrs) | 7.95 | 5.09 | 0.08 |  | 6.32 | 8.51 | 0.09 |  | 10.28 | 0.0 | ─ |
| Cold Ischemia (hrs) | 19.55 | 28.38 | 0.004 |  | 22.85 | 21.68 | 0.66 |  | 19.51 | 28.83 | 0.009 |
| Total Ischemia (hrs) | 31.04 | 35.60 | 0.07 |  | 32.07 | 33.66 | 0.56 |  | 32.44 | 32.82 | 0.91 |
| Outcomes |  |  |  |  |  |  |  |  |  |  |  |
| %CD34+ Viability | 79.33 | 76.01 | 0.57 |  | 80.17 | 73.84 | 0.29 |  | 72.75 | 89.86 | 0.0001 |
| CFU-TOTAL/10^5^ TNC | 222.58 | 325.75 | 0.36 |  | 341.29 | 97.44 | 0.02 |  | 100.16 | 659.00 | <0.0000 |
| CFU-GM/10^5^ TNC | 28.38 | 64.31 | 0.04 |  | 50.46 | 18.03 | 0.04 |  | 18.52 | 94.85 | <0.0000 |

^a^ Significance test cannot be performed because Facility B processed only vertebrae.

VB = Vertebral Body, IL = Ilia

**Table S5.** %CD34+ Beta Regression Model. The model shows the effects of warm ischemia, body cooling, and cold ischemia, on the percentage of viable CD34+ cells, controlling for the influence of other covariates.

|  | Beta Regression Model | | | |  | Leave-One-Out Bootstrap Cross-Validation | | | | | |
| --- | --- | --- | --- | --- | --- | --- | --- | --- | --- | --- | --- |
| Predictor | Coefficient | Standard Error | p | Odds  Ratio |  | Coefficient | Standard Error | Z | p | 95% C.I. | |
| β_0_ Constant | 3.11504 | 0.71 | <0.0001 |  |  | 3.15149 | 0.86 | 3.66 | <0.0001 | 1.465 | 4.838 |
| β_1_ Experience | -0.02482 | 0.02 | 0.09 | 0.9755 |  | -0.02282 | 0.02 | -1.05 | 0.29 | -0.065 | 0.020 |
| β_2_ Facility x Experience | 0.03203 | 0.02 | 0.07 | 1.0325 |  | 0.03023 | 0.02 | 1.51 | 0.13 | -0.009 | 0.070 |
| β_3_ Bone Type (VB=1) | 0.22102 | 0.26 | 0.40 | 1.2473 |  | 0.21252 | 0.28 | 1.21 | 0.22 | -4.225 | 17.996 |
| β_4_ Warm Ischemia (hrs)^a^ | -0.03423 | 0.05 | 0.52 | 0.9663 |  | -0.03775 | 0.06 | -0.63 | 0.53 | -0.156 | 0.080 |
| β_5_ Body Cooling (hrs) | -0.16779 | 0.05 | 0.002 | 0.8455 |  | -0.17062 | 0.06 | -2.67 | 0.008 | -0.296 | -0.045 |
| β_6_ Body Cooling Squared | 0.00592 | 0.003 | 0.03 | 1.0059 |  | 0.00609 | 0.003 | 1.84 | 0.06 | 0.004 | 0.012 |
| β_7_ Cold Ischemia (hrs) | -0.09982 | 0.033 | 0.003 | 0.9050 |  | -0.10165 | 0.05 | -1.87 | 0.06 | -0.208 | 0.005 |
| β_8_ Cold Ischemia Squared | 0.00144 | 0.0005 | 0.005 | 1.0014 |  | 0.00146 | 0.001 | 1.27 | 0.20 | -0.0008 | 0.004 |

Likelihood Ratio Chi-Square _(8)_ = 25.20, p = 0.001, AIC = -79.07

^a^ Interpretation: For Warm Ischemia the odds ratio is obtained from the regression coefficient as e^β^ = e^-0.03423^ = 0.966. The odds ratio indicates that each one-hour increase in warm ischemia reduces the average percentage of viable CD34+ cells to 96.6% of its previous value.

**Table S6.** CFU-Total Linear Regression: Effects of warm ischemia, body cooling, and cold ischemia on number of CFUs/10^5^ TNC controlling for the influences of facility, experience (number of cases processed), and bone type.

|  | Linear Regression Model | | |  | Leave-One-Out Bootstrap Cross-Validation | | | | | |
| --- | --- | --- | --- | --- | --- | --- | --- | --- | --- | --- |
| Predictor | Coefficient | Standard Error | p |  | Coefficient | Standard Error | Z | p | 95% C.I. | |
| β_0_ Constant | 160.6034 | 272.50 | 0.56 |  | 146.9639 | 242.57 | 0.61 | 0.54 | -328.46 | 622.39 |
| β _1_ Experience | 2.60499 | 7.54 | 0.73 |  | 2.89051 | 5.84 | 0.49 | 0.62 | -8.56 | 14.34 |
| β _2_ Facility x Experience | 5.36988 | 6.46 | 0.41 |  | 4.84149 | 5.55 | 0.87 | 0.38 | -6.03 | 15.72 |
| β _3_ Bone Type (VB=1) | 206.9969 | 90.04 | 0.025 |  | 188.5609 | 86.93 | 2.17 | 0.03 | 18.18 | 358.94 |
| β _4_ Warm Ischemia (hrs)^a^ | -3.73481 | 19.28 | 0.85 |  | -0.74672 | 28.98 | -0.03 | 0.98 | -57.54 | 56.05 |
| β _5_ Body Cooling (hrs) | -82.49506 | 18.92 | 0.00005 |  | -81.81929 | 20.37 | -4.02 | <0.0001 | -121.74 | -41.89 |
| β _6_ Body Cooling Squared | 2.95994 | 0.92 | 0.002 |  | 2.95785 | 0.97 | 3.06 | 0.002 | 1.06 | 4.85 |
| β _7_ Cold Ischemia (hrs) | 9.55975 | 12.53 | 0.45 |  | 10.27626 | 14.74 | 0.70 | 0.49 | -18.61 | 39.17 |
| β _8_ Cold Ischemia Squared | -0.12535 | 0.18 | 0.48 |  | -0.12796 | 0.25 | -0.50 | 0.61 | -0.63 | 0.37 |

Model F_(8,58)_ = 6.51, p = 0.000005, R^2^ = 0.473, R^2^adj = 0.40

^a^ Interpretation: The linear regression coefficient associated with Warm Ischemia indicates that each one-hour increase in warm ischemia reduces the number of CFUs by -3.73 x 10^5^ cells. At the average values of warm ischemia (2.99 hours), body cooling (7.81 hours), and cold ischemia (23.14 hours), the expected CFU yield is 93/10^5^ cells. With body cooling and cold ischemia held constant at their averages, a one-hour increase in warm ischemia from 2.99 hours to 3.99 hours would reduce the expected CFU yield from 93 x 10^5^ cells to 83.89 x 10^5^ cells (i.e., 93 – 9.11 = 83.89).

**Table S7.** CFU-GM Linear Regression: Effects of warm ischemia, body cooling, and cold ischemia on number of CFUs/10^5^ TNC controlling for the influence of bone type.

|  | Linear Regression Model | | |  | Leave-One-Out Bootstrap Cross-Validation | | | | | |
| --- | --- | --- | --- | --- | --- | --- | --- | --- | --- | --- |
| Predictor | Coefficient | Standard Error | p |  | Coefficient | Standard Error | Z | p | 95% C.I. | |
| β_0_ Constant | 88.3589 | 27.92 | 0.002 |  | 89.92144 | 28.05 | 3.17 | 0.002 | 33.95 | 143.89 |
| Β_1_ Bone Type (VB=1) | 16.71592 | 14.00 | 0.24 |  | 16.68794 | 12.58 | 1.33 | 0.18 | -7.97 | 41.35 |
| Β_2_ Warm Ischemia (hrs) | -7.19329 | 3.24 | 0.03 |  | -7.26995 | 2.70 | -2.69 | 0.007 | -12.57 | -1.97 |
| Β_3_ Body Cooling (hrs) | -5.24410 | 1.16 | 0.00003 |  | -5.28080 | 1.17 | -4.52 | <0.0001 | -7.57 | -2.99 |
| Β_4_ Cold Ischemia (hrs) | 0.10750 | 0.61 | 0.86 |  | 0.09875 | 0.53 | 0.18 | 0.85 | -0.95 | 1.15 |

Model F_(4,61)_ = 7.77, p < 0.00001, R^2^ = 0.338, R^2^adj = 0.294

**References**

1. Ferrari SLP, Cribari-Neto F. Beta regression for modeling rates and proportions. *Journal of Applied Statistics*. 2004, 31(7):799-815
2. Harrel Jr, F.E., *Regression modeling strategies with applications to linear models, logistic regression, and survival analysis*. 2nd ed. Springer Series in Statistics. 2001, New York: Springer. 582
